# Réparation juridique en dommage corporel de l’insuffisance antéhypophysaire post-traumatique

**DOI:** 10.11604/pamj.2017.28.277.12635

**Published:** 2017-11-28

**Authors:** Mohamed Mahjoub, Maher Jedidi, Zied Mezgar, Tasnim Masmoudi, Mongi Zhioua, Koussay El Euch, Mansour Njah

**Affiliations:** 1 Sevice d’Hygiène Hospitalière CHU Farhat Hached Sousse, Tunisie; 2 Service de Médecine Légale CHU Farhat Hached, Sousse, Tunisie; 3 Service des Urgences CHU Farhat Hached, Sousse, Tunisie; 4 Service de Medecine Légale CHU Charle Nicolle Tunis, Tunisie; 5 Service d’Endocrinologie CHU Farhat Hached, Sousse, Tunisie

**Keywords:** Insuffisance antéhypophysaire, post-traumatique, accident de circulation, imputabilité, réparation juridique, dommage corporel, Anterior pituitary insufficiency, road accident, imputability, legal redress, physical injury

## Abstract

L'insuffisance antéhypophysaire post-traumatique (IAHPT) est une pathologie exceptionnelle mais de réalité certaine résultant des lésions ischémiques lors des traumatismes crâniens (TC) sévères. L'objectif est de préciser à partir d'une étude de cas les critères d'imputabilité de l'IAHPT suite au (TC) ainsi que les spécificités relatifs à sa réparation juridique. C'est une étude médico-légale d'un cas d'IAHPT, diagnostiqué et suivi au service d'endocrinologie et de médecine légale du CHU de Sousse (Tunisie). Il s'agit d'une femme âgée de 45 ans, sans antécédents pathologiques (6 gestes, 4 parités et 2 avortements) ayant un cycle menstruel régulier, sans notion d'accouchement hémorragique, qui a été victime d'un accident de la voie publique (piétonne, heurtée puis renversée par une voiture) occasionnant un TC avec point d'impact occipital sans perte de connaissance initiale; ayant présenté trois ans après l'accident, une hypothyroïdie. L'exploration hormonale rapporte l'atteinte de tous les autres axes. L'exploration neuroradiologique retrouve une intégrité de l'hypophyse et de la tige. Le diagnostic définitif est l'IAHPT. L'expertise médicale (faite 4 ans après l'accident) a conclue à l'imputabilité de l'IAHPT à l'accident. Le taux d'incapacité partielle permanente IIP en droit commun a été évalué à 25%. L'IAHPT est un diagnostic d'élimination. L'évaluation du dommage corporel doit tenir compte des symptômes résiduels, contraintes thérapeutiques et répercussions sur l'activité quotidienne et professionnelle. L'évolution sous hormonothérapie de substitution est souvent favorable, cependant, elle peut être émaillée de complications, d'où l'obligation d'établir des réserves préservant ainsi le droit du patient à une nouvelle révision.

## Abstract (en)

Post-traumatic anterior pituitary insufficiency (PTAPI) is a rare but established disease caused by ischemic lesions after severe cranial trauma. This case study aims to highlight the criteria for imputability of PTAPI to a cranial trauma as well as the specific principles of legal redress in relation to this disease. We conducted a forensic study of a patient with diagnosed PTAPI followed up in the Division of Endocrinology and Legal Medicine at the University Hospital, Sousse (Tunisia). The patient included in the study was a 45-year old woman with no particular past medical history. (6 gynaecological procedures, 4 parities and 2 abortions) having regular menstrual cycles, without a history of hemorrhagic childbirth, who had been involved in a road accident (pedestrian got hit by a car) causing occipital trauma without initial knowledge loss. She developed hypothyroidism three years after the accident. The hormonal assessment showed the involvement of all the other axes. Neuroradiological examination objectified pituitary gland and pituitary stalk integrity. The definitive diagnosis of PTAPI was made. Medical examination (performed 4 years after the accident) concluded that PTAPI was imputable to the accident. Permanent partial disability rate according to ordinary law has been assessed at 25%. PTAPI diagnosis is made by elimination. The evaluation of physical injury should be based on residual symptoms, on therapeutic constraints and on its impact on daily and professional activity. Patient's evolution under hormone replacement therapy is often favorable, however, complications may occur, hence the necessity to establish these complications in order to preserve the right of the patient to underwent new treatments.

## Introduction

La pathologie endocrinienne post-traumatique est très rare en clinique endocrinologique comme en pratique médico-légale, compte tenu de la bilatéralité fréquente des glandes endocrines, de leur situation anatomique souvent profonde, protégée par d'autres structures et de la nécessité d'une lésion dépassant les 3/4 de la glande pour donner une insuffisance sécrétoire patente [1]. Le terme de « post-traumatique » doit être entendu dans son sens le plus large, c'est-à-dire un « état créé par l'effet d'une violence externe sur l'organisme », donc cela suppose que le mode d'action soit physique. Par ailleurs, il faut distinguer parmi les endocrinopathies post-traumatiques celles créées de toutes pièces, *de novo*, par le traumatisme, de celles *révélées* ou *aggravées* par un traumatisme. Cette distinction est fondamentale pour la notion *d'état antérieur* qui est très importante pour l'évaluation du dommage en droit commun et pénal. L'insuffisance anté-hypophysaire post-traumatique (IAHPT) est, sans doute, une pathologie exceptionnelle mais de réalité certaine. Sa rareté vient, particulièrement, du fait que les syndromes déficitaires qu'elle engendre ne sont pas de manifestations immédiates mais à distance du traumatisme et sont parfois rapportés à tort à d'autres pathologies. Ainsi, le délai écoulé entre le traumatisme et le diagnostic est assez long et la cause est souvent évoquée à postériori devant des carences hormonales patentes pourvu que la relation de causalité et l'imputabilité soient établies et prouvées entre le traumatisme causal et la symptomatologie présentée [2]. Cette complication importante du traumatisme crânien, devrait pouvoir être bien diagnostiquée et étiquetée médico-légalement. A partir d'une observation médico-légale de l'IAHPT crânien, nous discuterons la physiopahogénie, les circonstances traumatiques, les critères à retenir pour admettre l'imputabilité au traumatisme de l'IAHPT ainsi que les spécificités la réparation juridique de cette affection.

## Patient et observation

Il s'agit d'une patiente âgée de 45 ans qui présente comme antécédents familiaux un père diabétique non insulino-dépendant et des antécédents personnels se résumant a six Gestes gynécologiques, quatre Parités et deux avortements (4 enfants vivants: 1 garçon et 3 filles avec deux fausses couches spontanées survenant intercallerement aux naissances des 3 filles). Les accouchements sont normaux par voie basse avec des suites du post partum simples. Aucune notion d'antécédent d'accouchement hémorragique n'est retrouvée, le poids des naissances est normal, le cycle menstruel est régulier, aucune notion d'antécédents personnels de diabète ou de HTA (hypertension artérielle), de prise de médicament iodée ou d'irradiation cervicale ne sont retrouvés. Elle a été victime d'un accident de la circulation (étant piétonne qui aurait été heurtée puis renversée par une voiture), elle a présenté suite à cet accident un traumatisme crânien avec point d'impact occipital sans perte de connaissance initiale (hématome occipital de 4cm de diamètre sans plaie du cuir chevelu) et une fracture complète non déplacée tibio-péronienne droite. Elle a été hospitalisée durant 24 heures pour surveillance aux urgences. Elle a bénéficiée, entre autre, d'une immobilisation plâtrée du membre inférieur droit. Elle n'a pas eu d'hospitalisation au service des urgences devant l'absence des signes d'appel neurologiques avec un repos de quarante cinq jours (45j) au certificat médical initial. Elle aurait bénéficié de plusieurs consultations de suivi en orthopédie, avec prescription de traitement antalgique et anti-inflammatoire puis une ablation du plâtre (a sept semaines) avec indication de trois séances de rééducation fonctionnelle par semaine durant quatre semaines.

**Figure 1:**
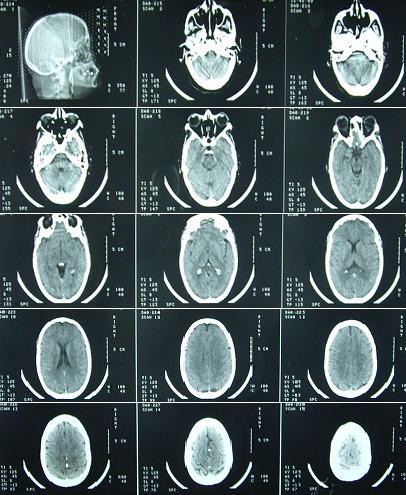
Tomodensitométrie cérébrale pratiquée trois années après le traumatisme crânien qui ne montre aucune lésion cérébrale en faveur de l’insuffisance antéhypophysaire

La patiente décrit depuis dix mois suivant l'accident une asthénie, une pâleur cutanéo-muqueuse, des céphalées, une frilosité, une peau sensible fine, des bourdonnements d'oreille, une intolérance au jeune, des troubles du cycle menstruel depuis une année avec une aménorrhée totale depuis huit mois sans notion de bouffée de chaleur et une chute de cheveux avec une dépilation axillaire. Trois ans après le traumatisme crânien et devant l'aggravation progressive de l'asthénie tendant vers les malaises retentissant sur les activités de la vie quotidienne, elle a consulté auprès d'un neurologue qui lui a fait pratiquer une TDM cérébrale (Figure 1) qui est revenue normale (Absence d'anomalies de visualisation de la tige pituitaire, pas d'hypoplasique ou d'ectopique des lobes anté-hypophysaire ou post-hypohyse) puis auprès d'un interniste qui a suspecté une hypothyroïdie et lui a fait pratiquer un bilan thyroïdien concluant à une hypothyroïdie centrale suite à la quelle le reste du bilan objective une insuffisance corticotrope, gonadotrope et lactotrope chez une multipare âgée de 48 ans. La patiente rapporte une nette amélioration de sa symptomatologie suite aux traitements hormonaux substitutifs qu'elle reçoit (hydrocortisone 10: 4 comprimés par jour, L thyroxine 100: un comprimé et demi par jour). Elle a été, par suite, admise au service d'endocrinologie pour exploration d'une hypothyroïdie centrale avec des diagnostics étiologiques évoqués penchant vers soit les hypophysites auto-immunes ou lymphocytaires soit l'insuffisance hypophysaire post traumatique. Le diagnostic définitif d'IAHPT est un diagnostic d'élimination, il a été retenu devant le contexte circonstanciel (AVP avec traumatisme crânien), la symptomatologie présentée par la patiente, le délai d'apparition des signes cliniques, les bilans endocrinologiques pratiqués et suite à l'élimination de tous les autres diagnostics étiologiques. L'expertise médicale a eu lieu cinq ans après l'accident et l'expert a conclu à l'imputabilité de l'insuffisance anté-hypophysaire à l'accident (insuffisance anté-hypophysaire post traumatique). Le taux d'I.I.P en droit commun a été évalué à 25%.

## Discussion

**L'imputabilité**: Dans notre observation, les troubles se sont manifestés après un délai relativement long suivant l'accident (dix mois) et le traumatisme possédait une composante crânienne. Par ailleurs, notre patiente n'avait aucun antécédent pathologique notable. Les troubles endocriniens qu'elle a présentés ont nécessité un traitement hormonal substitutif à vie. Le problème de discussion médico-légale soulevé par les endocrinopathies post-traumatiques est délicat. Il nécessite de justifier le dommage physique puis de l'estimer [3, 4].

**Realite du dommage**: *Affirmer la réalité du dommage physique, c'est porter un diagnostic certain de maladie endocrinienne*, sur les données cliniques et paracliniques, l'exploration humorale et hormonale, statique et dynamique, radiographies du crâne, EEG (electro-encéphalographique), tomodensitométrie, etc. Seule la synthèse minutieuse de tous ces éléments permettra d'affirmer l'authenticité et la nature de l'endocrinopathie [2, 5].

**Realite du tramatisme**: Si un traumatisme *direct* sur une glande endocrine a en quelque sorte, une réelle valeur de force de frappe juridique, le rôle des traumatismes *indirects,* même à distance, ne doit pas être minimisé. Dans ce cadre, à côté du traumatisme physique et dans la même perspective, le traumatisme *psycho-affectif émotionnel* a, d'un point de vue endocrinologique, la même valeur déterminante, précisément par le relais d'une perturbation fonctionnelle hypothalamique [3, 6].

**Relation de cause a effet**: Elle repose sur *L'élimination de toute coïncidence*: l'anamnèse, l'exploration clinique et paraclinique permettent de faire la part, dans les troubles observés, d'une autre cause responsable ou d'un état antérieur pathologique ou encore d'une prédisposition constitutionnelle génétique. Ainsi, *l'enchaînement chronologique des troubles endocriniens et du traumatisme* dont l'interprétation doit être nuancée: si les premiers signes endocriniens apparaissent *immédiatement* ou peu de temps après le traumatisme, l'enchaînement est indiscutable; s'ils apparaissent à retardement, voire tardivement, la preuve de cet enchaînement est plus difficile à établir. Or, la constitution retardée de troubles endocriniens est fréquente car elle nécessite la titularisation des lésions centrales responsables, certaines d'entre elles pouvant être de constitution lente (cal osseux hypertrophique, arachnoïdite, cicatrice), puis la dégénérescence ou la destruction du parenchyme glandulaire dont l'atteinte doit être très étendue pour avoir une traduction objective. Finalement, pour admettre le rattachement d'un traumatisme à une affection déterminée et démontrer qu'il en a été la cause génératrice et provocatrice, sept conditions médicolégales doivent se trouver réunies: se sont les 7 conditions de Simonin [2, 6].

### Validation des sept conditions de simonin

**L'absence d'état antérieur**: L'imputabilité d'un dommage à un fait traumatique ne peut être admise qu'en *l'absence d'un état antérieur pathologique*. L'existence de ce dernier ferait entrer le processus dans un mécanisme d'aggravation plutôt que de genèse initiale. Par ailleurs, le médecin expert doit accorder toute l'importance nécessaire à l'étape de recherche d'un état antérieur. Il doit être certain et doit prouver que le patient est indemne de l'insuffisance antéhypophysaire avant l'accident présumé causal [7]. Dans notre étude, ce critère a été validé puisque la patiente ne présentait aucun antécédent pathologique personnel particulièrement d'endocrinopathie à l'exception d'un diabète non insulinodépendant chez son père.

**La réalité du traumatisme**: La fréquence réelle des hypopituitarismes post-traumatiques est d'estimation assez difficile du fait de la sévérité des traumatismes en cause et de la sous estimation de cette pathologie. L'amélioration de la survie des traumatisés crâniens graves grâce aux techniques modernes de réanimation pourrait avoir pour effet à l'avenir, d'augmenter la fréquence réelle de ces cas. Le plus souvent, il s'agit de sujets de sexe masculin, d'âge jeune, ayant subi un traumatisme grave. Nous retrouvons comme cause les plus fréquemment décrites par ordre de fréquence les: accidents de la voie publique et de travail, Chutes, Traumatismes par balles ou par armes tranchantes ou par bombes et explosifs, Enfants Battus et Accident de parachute. Les lésions responsables sont variables: contusions directes, lésions dégénératives par ischémie, compression par héma¬tome, arachnoïdite, méningite séreuse de la base. Ces différentes lésions succèdent le plus souvent à une fracture de la base du crâne ou à une plaie par balle, ou à une commotion cérébrale lors d'une explosion (Lehman) [2, 3, 8]. Dans notre observation, le traumatisme est bien réel et multifocale avec une composante crânienne et un point d'impact occipital associé à un hématome de 4cm de grand axe sans plaie du cuir chevelu.

**L'intensité**: Comme argument de la sévérité du traumatisme initial, nous retrouvons que le nombre de fractures faisant suite à l'accident est estimé à environ 60% des cas et que ces fractures touchent le plus souvent: la base du sphénoïde, suivis par les fractures des os temporal et frontal et plus rarement, les fractures du squelette facial ou des os temporal et occipital. Il est rapporté dans la littérature, les fractures de la base du crâne et les lésions hypophysaires qui sont occasionnées, le plus souvent, par des coups reçus de derrière et d'en bas [6]. Le deuxième argument en faveur de la sévérité du traumatisme initial peut être rapporté par la présence ou non du coma en effet, dans 35% des cas décrit dans la littérature, il n'y a pas de coma ou bien qu'on ne le sait pas alors que dans le nombre de cas restant (soit 65% des cas décrite) le coma existe et sa durée varie de quelques minutes (10% des cas) à plusieurs jours (32% des cas) [2, 3, 8, 9]. Notre patiente étant piétonne, elle a été heurtée puis renversée par une voiture.

**La concordance de siège**: La règle en imputabilité, est qu'un dommage doit apparaître en principe, au niveau de la région du corps objet du traumatisme. L'IAHPT peut, toutefois, se voir, aussi en cas de traumatisme indirect du crâne tel que lors du traumatisme du rachis cervical qui peut entraîner un mouvement de va-et-vient brusque de celui-ci suite à la décélération inopinée succédant l'accélération. L'insuffisance antéhypophysaire secondaire à une *ischémie sévère* de l'hypophyse, elle-même due à un état de choc par hémorragie interne grave, est précisément celle invoquée par Sheehan à l'origine de l'insuffisance antéhypophysaire globale qu'il a décrite dans les suites des accouchements hémorragiques. Ainsi, contrairement au diabète insipide post-traumatique, on peut donc observer, dans un nombre non négligeable de cas, une insuffisance antéhypo¬physaire globale secondaire à un traumatisme à distance de la sphère céphalique, en dehors de tout traumatisme direct, dès qu'il y a eu état de choc important avec spoliation sanguine (Kerkhoven) [2-4, 6-8]. Dans notre étude ce critère a été validé puisqu'au point d'impact occipitale, la patiente a présenté un hématome de 4cm de diamètre de grand axe non extériorisé.

**Le délai logique entre traumatisme et dommage**: Il est bien établi, en réparation du dommage corporel et concernant ce critère, que plus ce délai est court, plus le lien de causalité ne sera étroit, mais, ce critère est en fait, difficile à uniformiser puisque le délai d'apparition d'un trouble varie selon la nature de ce trouble. C'est le cas de l'insuffisance antéhypophysaire post traumatique où un délai de latence peut atteindre quelques années après un traumatisme. La longueur de ce délai n'exclut pas l'imputabilité [6]. Bien que le traumatisme initial soit sévère, il existe souvent un retard considérable au diagnostic qui est en moyenne de 2 ans avec un maximum de 35 ans et exceptionnellement il est de 10 jours. L'avis de l'expert en matière d'endocrinopathie post-traumatique, ne devra jamais être demandé de façon précoce, qu'il devra réserver son jugement en demandant un certain délai supplémentaire, s'il le juge opportun, pour pouvoir apprécier réellement l'importance du syndrome endocrinien qui peut se constituer petit à petit et surtout son caractère irréversible, définitif [2, 6]. Dans notre observation, le début de la symptomatologie remonte a quelques mois après l'accident (dix mois) par contre le diagnostic finale n'a été retenu qu'après authentification complète du tableau clinique de l'insuffisance anté-hypophysaire devenant totale donc trois après le traumatisme.

**La continuité des symptômes**: Les symptômes et la gêne fonctionnelle résiduelle doivent observer une évolution continue entre le traumatisme initial et l'état final. Il en est ainsi des douleurs post-traumatiques. Dans certaines circonstances, la gêne peut réapparaître après une phase silencieuse (syndrome post-intervallaire) sans que l'imputabilité n'en soit éliminée. Dans l'insuffisance antéhypophysaire post-traumatique, ce hiatus silencieux entre le traumatisme crânien initial et l'apparition des manifestations cliniques est illustré au bout, parfois, de plusieurs années après la survenue du traumatisme causal [2, 6]. Ce critère a été validé dans notre observation puisque il y a une continuité sans interruption de la symptomatologie présentée par la patiente.

**La pathogénie scientifiquement admise et une certitude du diagnostic**: Les symptômes initiaux les plus fréquents sont ceux de l'hypogonadisme, notamment la perte du libido, l'aménorrhée et l'impuissance. Ces symptômes sont, le plus souvent, attribués à un syndrome subjectif post traumatisme crânien et sont parfois ignorés pendant plusieurs années. Habituellement les signes cliniques sont ceux d'une insuffisance anté-hypophysaire globale. Dans sa forme complète et évoluée, elle réalise le triple tableau d'une insuffisance thyroïdienne, surrénale et gonadique [2, 8, 10, 11]. Mais du fait de l'origine primitive « haute » de ces différentes insuffisances glandulaires périphériques, certaines manifestations sémiologiques prennent valeur toute particulière d'orientation diagnostique et étiologique, à savoir: l'absence de sudation, de bouffées de chaleur insolite chez une femme ménopausée; l'absence de pigmentation au cours d'une insuffisance surrénale lente, avec même un aspect particulièrement dépigmenté de l'ensemble de la peau et des zones normalement pigmentées telles que les mamelons ou les zones génitales; l'absence de myx'dème vrai au cours d'une insuffisance thyroïdienne [3, 12]. Finalement on admet que toute aménorrhée « froide, sèche », c'est-à-dire sans bouffées de chaleur, doit faire soupçonner chez la femme non ménopausée une lésion hypophysaire tumorale ou ischémique, post-traumatique ou non [13].

En ce qui concerne l'insuffisance gonadique, une place à part doit être réservée à deux problèmes particuliers: l'impuissance ou la frigidité: relativement fréquentes au décours des traumatismes crâniens. Mais il est bien difficile de leur assigner toujours une origine hormonale. En effet, l'administration substitutive des hormones sexuelles, mâles ou femelles selon les cas, ne résout pas toujours le problème; le problème de la stérilité: chez la femme, la stérilité est dans la très grande majorité des cas secondaire à une anovulation d'origine hypothalamo-hypophysaire. En revanche, chez l'homme, la stérilité par atteinte de la spermatogenèse sous forme d'oligo-astheno-spermie, soit même d'azoospermie, semble beaucoup plus redoutable, faute de moyens thérapeutiques efficaces susceptibles de la corriger [2, 14, 15]. Les manifestations neuro-ophtalmologiques sont fréquentes, les plus communes sont: la cécité unilatérale ou bilatérale avec des déficits variables du champ visuel variant d'un scotome jusqu'à une hémianopsie homonyme. A moindre degré de fréquence l'atteinte des V-VI-VII paire crânienne. L'hémiparésie et l'hémianesthésie, l'anosmie, la cécité complète, quadriplégie spastique et l'atteinte des autres paires crâniennes sont beaucoup moins fréquentes [8]. Dans notre observation ce critère a été dûment validé puisque le diagnostic a été confirmé par la symptomatologie présentée, le bilan hormonal, l'imagerie par la TDM cérébrale, les circonstances traumatiques et l'élimination de tous les autres étiologies éventuelles.

### Conséquences médico-légales et évaluation du dommage

Il faut savoir que la détermination de cette incapacité secondaire à l'IAHPT est particulièrement difficile. L'expert doit s'efforcer d'avoir la meilleure maîtrise possible de la physio-pathogénie et des aspects clinico-biologiques de ces affections [2, 6].

**L'incapacite temporaire**: Deux éléments, souvent associés mais pouvant être retrouvés séparément, interviennent dans l'appréciation de l'incapacité temporaire: l'autonomie personnelle d'une part et les conséquences de l'accident sur l'activité professionnelle d'autre part. L'incapacité temporaire professionnelle ou non professionnelle résultant de l'IAHPT peut être soit partielle avec un certain degré d'indépendance du patient (reprise du travail avant la consolidation, modification temporaire du poste de travail, travail à temps partiel temporaire), soit totale avec une dépendance totale du patient (l'évaluation a postériori des dommages tiendra compte des hospitalisations et des durées de l'immobilisation en alitement du patient a domicile) [4].

**La date de consolidation**: Ce délai n'est pas toujours aisé à fixer et reste surtout pour cette affection post-traumatique rare, laissé à l'appréciation en son âme et conscience au médecin expert. Ce délai est très variable et est, en moyenne, de 6 mois à un an dans l'IAHPT. Il dépendra du génie évolutif de la maladie. Dans tous les cas, avant de déterminer le niveau de réparation, l'expert doit avoir toute la latitude et attendre un temps suffisant pour obtenir le meilleur équilibre possible de l'affection [16, 17].

**L'incapacite permanente: facteurs d'appréciation de l'incapacité permanente**

**Les contraintes thérapeutiques**: Les contraintes thérapeutiques sont classées en: contraintes légères: le seul fait pour le patient de consommer quotidiennement le traitement substitutif per os est en soi une contrainte, même si elle peut confiner à l'automatisme; contraintes modérées: le traitement médicamenteux substitutif est relativement lourd et est considéré comme une contrainte. Ajouté à cela le degré de gêne, variable selon les individus, liée aux effets secondaires; contraintes moyennes: suite à une instabilité clinique malgré le traitement substitutif quelque soit son étiologie nécessitant des contrôles médicaux réguliers et des bilans biologiques de contrôles rapprochés avec recours parfois à des régimes alimentaires spécifiques; contraintes graves: suite aux complications secondaires à un arrêt du traitement même transitoire et quelques soit son étiologie pouvant aboutir à des accidents hypoglycémiques, des collapsus ou plus gravement un coma hypopituitaire [2, 18].

**Les conséquences sur l'activité quotidienne de la vie courante**: Les gênes résultant de la restriction qualitative et/ou quantitative des activités quotidiennes du sujet et leur retentissement sur la vie courante sont: activité d'agrément (d'asthénie retentissant sur les performances sportive antérieures, voire l'impossibilité de les continuer), activité personnelle (fatigabilité et gêne de la vie quotidienne, déplacements contrariés. Interdiction de certains voyages), activités professionnelles (nécessitant, selon les besoins, une réorientation, une restriction des capacités de travail, une réadaptation du poste du travail voire même un reclassement) et des troubles sexuels.

**L'instabilité clinique éventuelle**: Il faut apprécier lorsque aucune amélioration nouvelle ne semble possible, la qualité de l'équilibre obtenu et les symptômes résiduels non totalement compensés par le traitement substitutif (la fréquence des hypoglycémies, les tendances spontanées au retard de croissance) [2].

**Les facteurs pronostics**: Le pronostic vital de l'insuffisance antéhypophysaire qui se posait autrefois, avec risque de mort subite par hypoglycémie, de collapsus cardiovasculaire hypovolémique, ne se pose plus actuellement depuis l'avènement de la corticothérapie substitutive. Avec une triple hormonothérapie substitutive, comblant les déficits thyroïdien, surrénal et gonadique, on peut redonner à l'individu un équilibre hormonal satisfaisant. Mais, même bien conduit, le traitement hormonal ne restaure pas la fonction endocrinienne dans son intégralité, dans la mesure où précisément l'auto-régulation n'est plus possible, obligeant à un traitement définitif sans interruption [2, 6].

**Les taux de l'incapacité permanente selon les régimes de réparation**

La détermination de l'IPP est délicate et nuancée, *elle* sera fonction de l'âge du sujet, du retentissement de la maladie endocrinienne sur la croissance, le développement génital, la musculature, de la possibilité de guérison vraie, de l'existence des séquelles et de la nécessité ou non d'un traitement substitutif définitif.

**En droit commun**: Pour l'insuffisance antéhypophysaire post traumatique, la société de médecine légale et de criminologie de France propose des taux allant de 10 à 30% et ce six mois à un an après la mise en 'uvre du traitement substitutif et en fonction de la qualité des résultats obtenus sur les signes clinique invalidants (asthénie, frilosité, stérilité, frigidité), des limitations de l'activité et de l'importance des contraintes thérapeutiques. Il faut tenir compte également de l'incapacité due aux complications présentes au moment de l'expertise [16, 17, 19].

**En accident de la voie publique (AVP)**: Le barème d'évaluation médico-légale des incapacités permanentes des victimes d'accidents de la circulation tel que décrit par la Loi n°: 2005-86 du 15 Aout 2005 suite à l'arrêté conjoint du ministre des finances et du ministre de la santé publique propose des taux allant de 10 à 30% et ce comme suit: six mois à un a après mise en 'uvre du traitement substitutif et en fonction de la qualité des résultats obtenus sur les signes cliniques invalidants (asthénie, frilosité, stérilité, frigidité), des limitations de l'activité et de l'importance des contraintes thérapeutiques [20].

**En accident du travail**: Le taux d'I.P.P est fixé par la commission médicale selon d'une part des critères d'ordre médical: la nature et la gravité de l'atteinte, l'état général, l'âge, les facultés physiques et mentales et d'autre part, d'un critère d'ordre professionnel: les aptitudes et la qualification professionnelle de la victime en tenant compte des données du barème indicatif d'invalidité permanente consécutive aux séquelles d'accident du travail et des maladies professionnelles. Le barème tunisien a fait l'objet de l'arrêté des Ministères de la Santé Publique et des Affaires Sociales du 10 janvier 1995. L'origine traumatique n'est retenue qu'une fois éliminée toute preuve d'existence d'insuffisance antéhypophysaire et notamment sur les résultats d'analyses disponibles. Selon la stabilité ou l'instabilité de l'équilibration thérapeutique, selon le degré de déficit et le résultat du traitement: 60 à 70% [21].

**Reparation des complications**: L'expert doit, de préférence, faire des réserves, quant à l'avenir: il n'y a pas d'insuffisance antéhypophysaire, si partielle ou si minime soit-elle, si bien traitée et si bien prise en charge soit-elle, qui ne se complique à plus ou moins longue échéance (tel que les troubles de la croissance et de la fertilité). Le traitement substitutif permet surtout de prévenir l'évolution spontanée vers les complications, à savoir les accidents hypoglycémiques, de collapsus ou plus gravement le coma hypopituitaire. Si l'insuffisance antéhypophysaire est imputable en totalité, le taux d'incapacité sera révisable en fonction de l'évolution de la maladie et des complications qui en découlent. L'expert doit mentionner la possibilité fréquente de complications tardives, sans cependant être prise en compte dans l'évaluation « actuelle » du taux d'incapacité [2, 6].

## Conclusion

L'IAHPT est une entité rarement décrite dans le domaine de la réparation juridique du dommage corporel et le médecin expert est exceptionnellement confronté aux problèmes des troubles endocriniens dans la pathologie séquellaire. L'analyse du lien de causalité n'est pas toujours aisée, l'évolutivité de la maladie et la grande disparité de la symptomatologie clinique rendent ce travail plus délicat. L'imputabilité médico-légale nécessite la confrontation des différents critères pour pouvoir se prononcer qu'il existe un faisceau d'argument suffisamment probant et pour admettre l'IAHPT séquellaire. L'évaluation du dommage corporel est très délicate dans le cadre d'endocrinopathie post traumatique et particulièrement dans l'insuffisance antéhypophysaire post traumatique qui mérite d'être signalé. Elle doit se faire au cas par cas en tenant compte des symptômes résiduels, des contraintes thérapeutiques et des répercussions sur les activités professionnelles et quotidiennes. La particularité de la consolidation médico-légale de l'insuffisance antéhypophysaire post traumatique, réside dans le fait qu'il n'y pas une fin de la thérapeutique active curatrice et le traitement hormonale de substitution est à vie. La décision de la consolidation, est un véritable moment sensible pour le médecin expert. Elle ne peut être fixée qu'après quelques années du traumatisme incriminé.

## Conflits d’intérêts

Les auteurs ne déclarent aucun conflit d'intérêts.
